# Assessing the Longitudinal Relationship Between Alcohol Use and Depression Among Older Adult Retirees

**DOI:** 10.1093/geroni/igaa057.1289

**Published:** 2020-12-16

**Authors:** Antonia Diaz-Valdes Iriarte, Christina Sellers

**Affiliations:** 1 Universidad Mayor, La Reina, Santiago, Region Metropolitana, Chile; 2 Simmons University, Boston, Massachusetts, United States

## Abstract

Alcohol use and depression are underrecognized problems affecting older adults, and its prevalence has increased for baby boomers compared to prior generations. Approximately 40% of people aged 65+, drink alcohol and approximately 19% experience. Additionally, the loss of important roles in life – such as being an employee, could negatively influence mental health at retirement. Although there is mixed evidence about the direction of the association between alcohol and depression in this population, and little is known about how retirement influence it. Generalized mixed models were performed to test the effect of retirement, alcohol use – drinks per day and binge drinking, and depressive symptoms among older adults. We draw data from the Health and Retirement study (n=11,164). Results suggest that being retired was associated with decreased depressive symptoms (b=-0.04, p<0.05) and increased drinks per day (b=0.01, p<0.05) and binge drinking (b=0.11, p<0.05). Each additional drink per day (b=-0.11, p<0.05) and binge drinking (b=-0.07, p<0.05) lead to decreased depressive symptoms. Similarly, increased drinking or binge drinking were associated to decreased depressive symptoms. Regular drinkers might increase their drinking to occupy the additional free time that comes with retirement, to alleviate anxiety and feelings of loneliness as they lost their working roles and they might have lost relatives, which might increase the craving for alcohol. Given the prevalence of depression and alcohol use, and its detrimental effects among older adults. Further research needs to be done to understands the particularities of this group and to generate interventions focused on prevention.

